# Supplementary material for: Plasmonic Nanolenses Produced by Cylindrical Vector Beam Printing for Sensing Applications
Source: Sci Rep. 2019 Dec 24;9:19750. doi: 10.1038/s41598-019-56077-8 (PMC6930225; doi:10.1038/s41598-019-56077-8)
Supplement: Supplementary file 1 — Supplementary Information [file 41598_2019_56077_MOESM1_ESM.pdf]

Supporting information for: “Plasmonic Nanolenses Produced by Cylindrical Vector Beam Printing for Sensing Applications.”

S.A. Syubaev[1,2], A.Yu. Zhizhchenko[1,2], D.V. Pavlov[1,2], S.O. Gurbatov[1,2], E.V. Pustovalov[1], A.P. Porfirev[3,4], S.N. Khonina[3,4], S.A. Kulinich[1,5], J.B.B. Rayappan[6], S.I. Kudryashov[7,8], and A.A. Kuchmizhak[1,2,*]

[1]Far Eastern Federal University, Vladivostok, Russia
[2]Institute of Automation and Control Processes, Far Eastern Branch, Russian Academy of Sciences, Vladivostok, Russia
[3]Samara National Research University, Samara, Russia
[4]IPSI RAS - Branch of the FSRC “Crystallography and Photonics” RAS, Samara, Russia
[5]Department of Mechanical Engineering, Tokai University, Hiratsuka, Kanagawa, Japan
[6]School of Electrical and Electronics Engineering, SASTRA Deemed University, Thanjavur, Tamil Nadu, India
[7]Lebedev Physical Institute, Russian Academy of Sciences, Moscow, Russia
[8]National Research Nuclear University MEPhI, Moscow, Russia
[*]alex.iacp.dvo@mail.ru

ABSTRACT

Here, we demonstrate a simple direct maskless laser-based approach for fabrication of back-reflector-coupled plasmonic nanorings arrays. The approach is based on delicate ablation of an upper metal film of a metal-insulator-metal (MIM) sandwich with donut-shaped laser pulses followed by argon ion-beam polishing. After being excited with a radially polarized beam, the as-prepared MIM configuration of the nanorings permitted to realize efficient nanofocusing of constructively interfering plasmonic waves excited in the gap area between the nanoring and back-reflector mirror. For optimized geometric parameters of such MIM structure, substantial enhancement of the electromagnetic near-fields at the center of the ring within a single focal spot with the size of $0.37\lambda^2$ can be achieved, which is confirmed by Finite Difference Time Domain (FDTD) calculations, as well as by detection of enhanced PL signal from adsorbed organic dye molecules. The simple large-scale and cost-efficient fabrication procedure used, along with relatively good tolerance to excitation beam misalignment, make the proposed structures promising for realization of various nanophotonic and biosensing platforms that utilize cylindrical vector beam as a pump source.

Contents

1 Details of the FDTD simulations 2

2 Laser printing of MIM nanorings with Gaussian-shaped beams 2

3 FDTD characterization of an isolated nanoring on a glass substrate pumped with CVBs 3

4 FDTD modeling of the MIM nanorings of variable diameter 4

5 FDTD modeling of the MIM nanorings with a nanoantenna 5

1 Details of the FDTD simulations

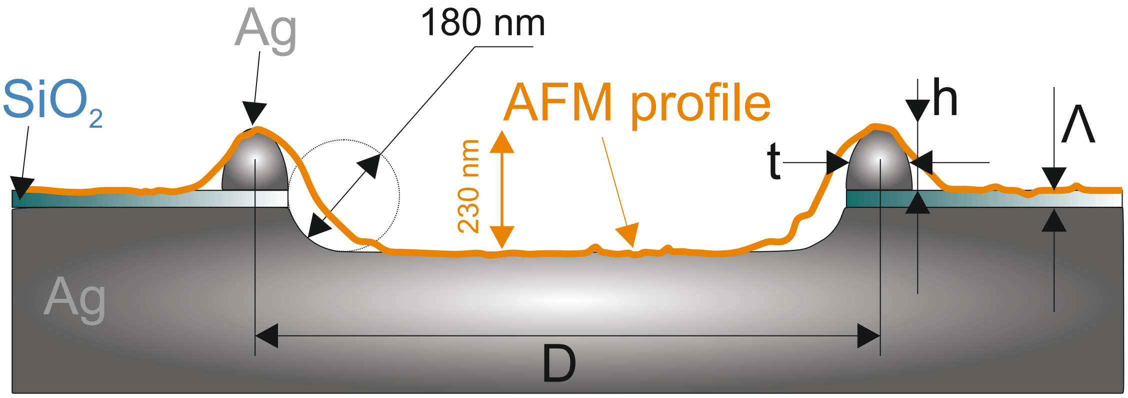

Figure 1. Schematic representation of the modeled MIM nanoring with an indication of all main geometric dimensions. Experimentally measured AFM profile is shown by orange curve. The deviation of the measured and modeled profile is caused by inaccuracy of the AFM profiling for abrupt height jumps with is confirmed by corresponding SEM imaging of the structures under study.

2 Laser printing of MIM nanorings with Gaussian-shaped beams

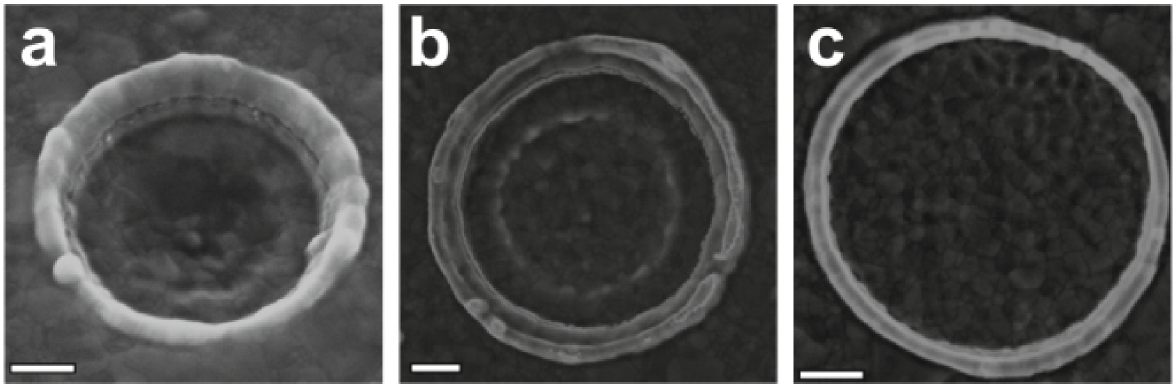

Figure 2. Representative SEM images of MIM nanorings produced using Gaussian-shaped (left and middle) and donut-shaped (right) single-pulse ablation of upper Ag film. Scale bar corresponds to 200 nm.

3 FDTD characterization of an isolated nanoring on a glass substrate pumped with CVBs

Here, we calculated excitation of an isolated Ag nanoring placed on a glass substrate (refractive index n=1.45) by a radially and azimuthally polarized CVB. For these calculations, we have used the geometrical parameters of the nanoring, which appeared to be optimal for the case of MIM geometry (see main manuscript). Figure S3 shows the normalized squared electric field amplitude $|E^2|/|E_0^2|$ calculated near the nanoring (top view) on a glass substrate upon excitation with either a radially (left) and an azimuthally (right) polarized CVB. As seen, radially polarized excitation can be coupled to plasmonic oscillations of the electron plasma localized in the nanoring walls resulting in 5-fold enhancement of the EM field around its surface. The central focal spot appears to be caused by the interference of the plasmon-mediated scattering waves. In a sharp contrast, the azimuthally polarized irradiation weakly couples to plasmon wave excitation in the nanorings resulting in negligible field enhancement.

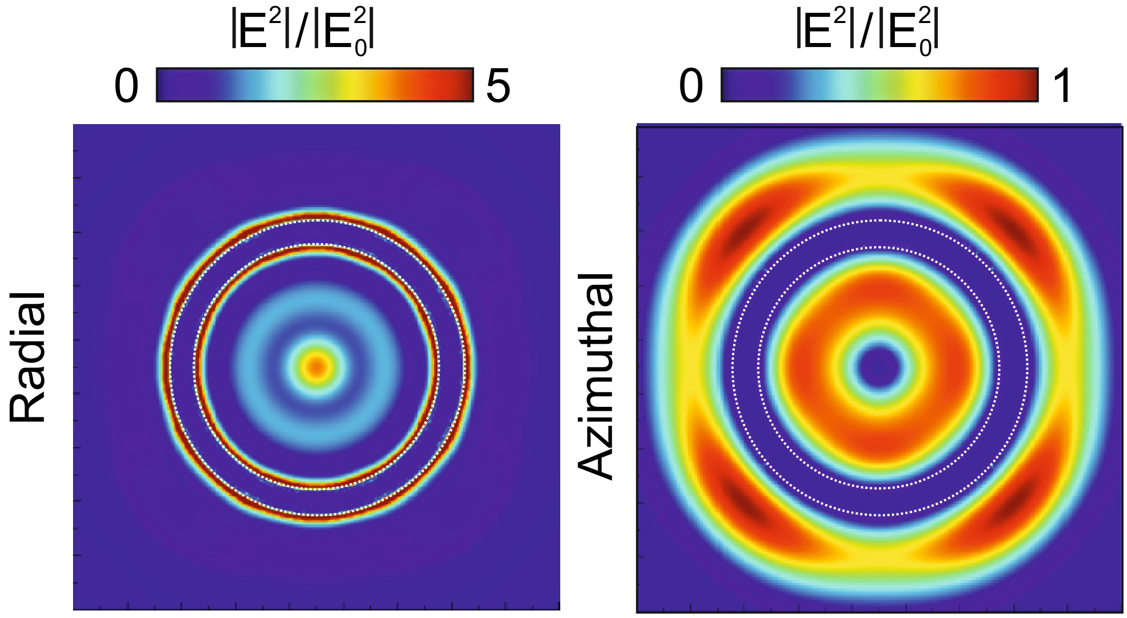

Figure 3. Normalized squared electric field amplitude $|E^2|/|E_0^2|$ calculated near the nanoring on a glass substrate upon excitation with radially (left) and azimuthally (right) polarized 1-μm diameter CVB at 532 nm excitation wavelength. Geometrical parameters of the modeled MIM nanoring are D= 1 μm, h=100 nm, t=100 nm. White circles highlight the geometry of the MIM nanoring. Maps are calculated in the plane situated 5 nm above the glass surface supporting the nanoring.

4 FDTD modeling of the MIM nanorings of variable diameter

Here, we modeled performance of the MIM nanorings having variable diameter D upon their excitation with a radially polarized CVB. For these simulation we took into account the experimentally defined range of available diameters of 900<D<1200 nm. Figure S4 shows the normalized squared electric field amplitude $|E^2|/|E_0^2|$ calculated in the central plasmonic "hot spot" versus the nanoring diameter D (h=100 nm, t=100 nm, Λ=25 nm). As seen, the maximal $|E^2|/|E_0^2|$ value is achieved at D=1 μm decreasing for any variation of the nanoring diameter within the modeled range. The substantial EM field enhancement can be explained in terms of constructive interference of the SP waves having $\lambda_{SP}=n_E\lambda$ (where $n_E=\sqrt{\varepsilon_m(\varepsilon_m+1)^{-1}}$ and ε_m is the permittivity of Ag. At λ=532 nm, the effective refractive index of the SP waves running at air-metal interface is $n_E \approx 1.04$ yielding in $\lambda_{SP} \approx 511$ nm. In this respect, from the first glance for interfering SP waves the the performance of the MIM nanoring versus its diameter D is expected to have periodic dependence with the periodic equal to λ_{SP}. However, more detailed studies are to be performed to clarify the dependence of maximal field enhancement versus MIM nanoring diameter, which value will be outside the range provided by the developed fabrication technique (900<D<1200 nm). These studies fall outside the scope of this paper.

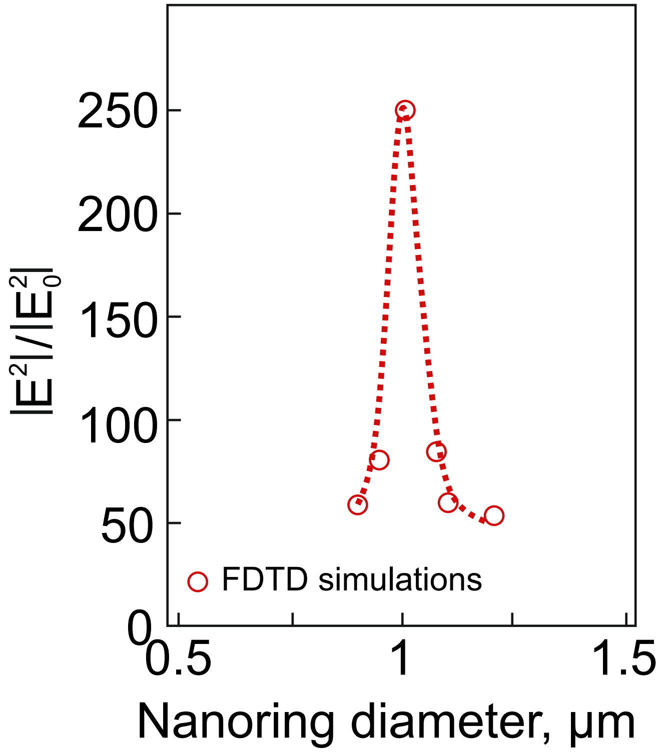

Figure 4. Calculated normalized squared electric field amplitude $|E^2|/|E_0^2|$ in the central plasmonic "hot spot" as a function of nanoring diameter D (red markers). Geometrical parameters of the modeled MIM nanoring are h=100 nm, t=100 nm and Λ=25 nm.

5 FDTD modeling of the MIM nanorings with a nanoantenna

Here, we calculated the performance of the optimized MIM nanoring with a 100-nm diameter Ag nanoparticle placed at the geometric center of the nanostructure. As seen, the converging SP waves can efficiently pump the gap plasmon mode in the nanoparticle-substrate system resulting in huge EM field enhancement as large as $|E^2|/|E_0^2| \approx 10^6$.

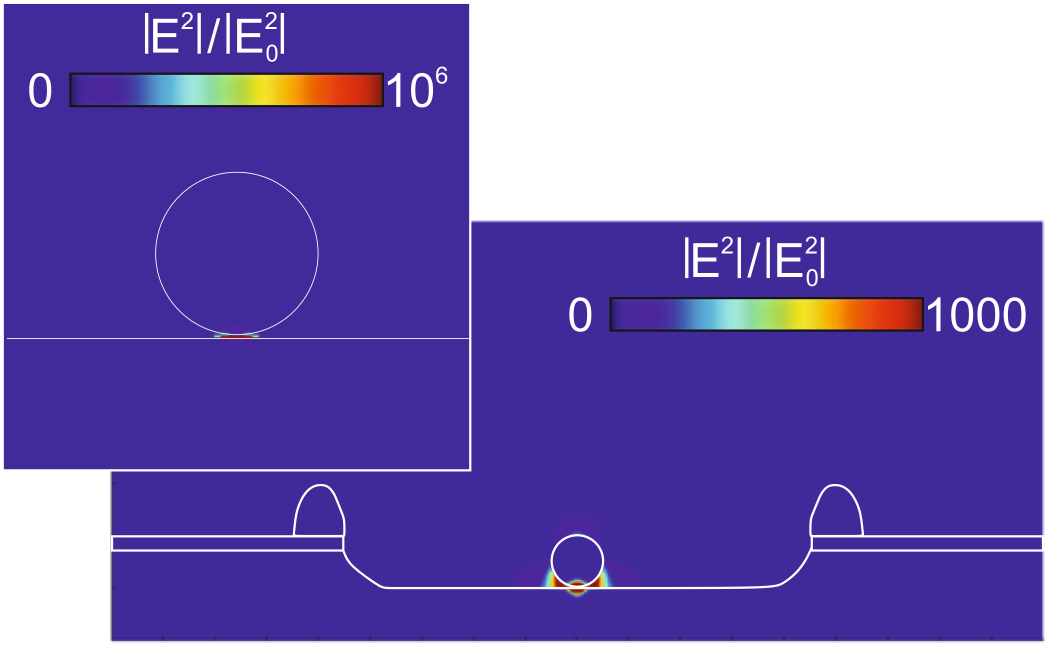

Figure 5. Normalized squared electric field amplitude $|E^2|/|E_0^2|$ calculated near the MIM nanoring with a spherical 100-nm diameter Ag nanoantenna upon excitation with radially polarized 1-μm diameter CVB at 532 nm excitation wavelength. Geometrical parameters of the modeled MIM nanoring are D= 1 μm, h=100 nm, t=100 nm. White curves highlight the geometry of the modeled structure.
